# Knowledge of prevention, cause, symptom and practices of malaria among women in Burkina Faso

**DOI:** 10.1371/journal.pone.0180508

**Published:** 2017-07-03

**Authors:** Sanni Yaya, Ghose Bishwajit, Michael Ekholuenetale, Vaibhav Shah, Bernard Kadio, Ogochukwu Udenigwe

**Affiliations:** 1 School of International Development and Global Studies, Faculty of Social Sciences, University of Ottawa, Ottawa, Ontario, Canada; 2 School of Medicine and Health Management, Tongji Medical College, Huazhong University of Science and Technology, Wuhan, Hubei, China; 3 The Women’s Health and Action Research Centre, Benin City, Nigeria; 4 Interdisciplinary School Health Sciences, University of Ottawa, Ottawa, Ontario Canada; 5 School of International Development and Global Studies, Faculty of Social Sciences, University of Ottawa, Ottawa, Ontario, Canada; Centro de Pesquisas Rene Rachou, BRAZIL

## Abstract

**Background:**

Malaria remains a major public health issue in most southern African countries as the disease remains hyper endemic. Burkina Faso continues to face challenges in the treatment of malaria, as the utilization of preventive measures remains low on a national scale. While it has been acknowledged that understanding women’s health-seeking behaviours, perception of malaria and its preventive measures will aid in the control of malaria, there is paucity of information on Knowledge, Attitudes and Practices among women in the reproductive age of 15–49 years in Burkina Faso. This study investigated women’s knowledge of malaria, attitudes towards malaria, and practices of malaria control in order to create a synergy between community efforts and governmental/non-governmental malaria control interventions in Burkina Faso.

**Methods:**

The analysis used data from the 2014 Burkina Faso Malaria Indicator Survey (MIS). In total 8111 women aged between 15–49 years were included in the present study. We assessed women’s knowledge about 1) preventive measures, 2) causes and 3) symptoms of malaria, as well as malaria prevention practices for their children and during pregnancy. The socio-demographic characteristics were considered for Age, Religion, Education, Wealth index, Number of household members, Sex of household head, Household possession of radio, TV and Received antenatal care. Data were analyzed using STATA, version 14. Associations between variables were tested using a Chi-square and logistic regression, with the level of statistical significance set at 95%.

**Results:**

A preponderant proportion of respondents were aged 15–29 years (mean age was 28.63±9.41). About three-quarters of the respondents had no formal education. An estimated two-third of the participants were of Islamic faith, while access to media and behavioural communication were generally poor. The level of knowledge was 53% for rural women and 68.2% for urban dwellers. In sum, there was 56.1% level of accurate knowledge of malaria among women in Burkina Faso. In the multivariable logistic regression, women in rural location had 40% reduction in the odds of having accurate knowledge of malaria when compared to urban women (aOR = 0.60; 95%CI: 0.52–0.68). The educational level was a key factor in the knowledge of malaria. The odds of having accurate knowledge of malaria increased as the educational level increased, hence, women with secondary and higher education had 29% and 93% increase in the odds of having accurate knowledge of malaria when compared to the women without formal education. Results indicate that antenatal care (ANC) services were major sources of information on malaria. Women who reportedly received ANC were 3.9 times more likely to have accurate knowledge of malaria when compared to those who did not utilize skilled ANC services (aOR = 3.90; 95%CI = 3.34–4.56).

**Conclusion:**

The overall knowledge of malaria prevention practices among a large proportion of women was found to be low, which implies that the knowledge about the prevention of malaria should be improved upon by both urban and rural dwellers. There is need for concerted behavioural communication intervention to improve the knowledge of malaria especially for rural dwellers regarding malaria prevention measures, causes and symptoms. Consistent efforts at providing relevant information by health organizations are needed to reduce and control incidences of malaria in the general public.

## Background

According to WHO estimates, 212 million global cases of malaria led to 429,000 global deaths in 2015 [1]. The burden was highest in the Sub-Saharan African region where 90% of the malaria cases and 92% of the malaria deaths occurred. Children under 5 years accounted for two thirds of deaths in this region [1]. The disease is severe in pregnant women and children under 5 years old [2,3]. Moreover, approximately 7 million cases of malaria leading to 15,000 deaths were reported in Burkina Faso in 2015 [1]. Of the country’s 16.2 million population, 80% reside in rural areas [4]. The effective treatment of malaria in rural populations in Sub-Saharan Africa is often impeded by poor housing quality, inadequate understanding of malaria causes and transmission, and preference for traditional treatments [5,6].

Food security challenges in Burkina Faso prompted the development of irrigation schemes, which promotes the proliferation of mosquitoes and malaria transmissions [7]. Malaria remains a major public health issue in Burkina Faso as the disease remains hyper endemic and stable [7]. In malaria-stable areas, residents can develop immunity to malaria and become asymptomatic; a vast majority of women with placental malaria are asymptomatic during pregnancy [4]. Persistent asymptomatic malaria infection has been viewed as beneficial to individuals as it reduces the risk of severe infection; however, it can lead to future transmissions [8,9]. Malaria is associated with high risk factors for maternal and neonatal morbidity and mortality, such as maternal anemia, preterm delivery and placental malaria, which leads to low birth weight babies [8,10,11].

Burkina Faso’s national malaria control program promotes the use of insecticide-treated bed nets (ITN) through their free distribution. The program also provides available and accessible artemisinin-based combined therapy (ACT) and free intermittent preventive treatment (IPT) for vulnerable groups particularly pregnant women. Furthermore, the country aligned strategies for malaria prevention with WHO’s recommendation and adopted the seasonal malaria chemoprevention (SMC) program for children under 5 years [4,7]. Indoor residual spraying (IRS) was also included as an additional preventive measure. Burkina Faso continues to face challenges in the treatment of malaria as the utilization of preventive measures remains low on a national scale [12,13]. Less than 1% of the population used IRS in 2014 and despite the equity in ITN possession among households, 25% of children were not sleeping under ITNs in 2014 [6]. Furthermore, there is a preference for self-treatment using local herbs, which leads to the delay in treatment at health facilities [4,14].

The goal of WHO to end the malaria epidemic by 2030 lies in its commitment to “continue to invest in changing people’s behaviour” [15]. A gendered approach to understanding the knowledge, attitudes and practices (KAP) of malaria and its control is necessary because gender norms influence labour, leisure and sleeping patterns, which invariably impacts exposures to mosquitoes. There are also gender dynamics in treatment-seeking behaviours and authority within households [16–19]. Women in particular are vulnerable to the disease especially during pregnancy. They are also responsible for home-based management of malaria among children under 5 years of age, who are highly vulnerable to malaria [20,21]. Women also serve as role models for their families in raising awareness and participating in malaria prevention and control.

The awareness of malaria vectors, mosquito behavioural pattern (biting and resting times) and breeding sites have been associated with the severity of malaria [22]. A study in Tanzania, with a majority of female participants, showed a poor understanding of mosquito behaviour pattern and breeding sites in an area with a high prevalence of malaria [23]. Little or no benefit of malaria control programs have been reported in areas where there is a lack of awareness of malaria control strategies [24–27]. Furthermore, women in the south-central district of Ethiopia were reported to have a high level of general knowledge on malaria, such as symptoms and treatment; however, their knowledge of causes of malaria and preventive measures were generally low [28]. Consequently, the use of preventive measures against malaria was low in households within the district [28]. Moreover, local concepts of illness affect choices regarding malaria treatment. In Burkina Faso, mothers' perception and interpretation of disease were reported to influence treatment choices for malaria [29]. Some manifestations of malaria were misdiagnosed by traditional methods and they were often self-treated at home. This shows that biomedical concept of malaria is not adequately understood. Moreover, traditional treatment of malaria delays effective biomedical treatment and can be fatal [29].

While it has been acknowledged that understanding women’s health-seeking behaviours, perception of malaria and its preventive measures will aid in the control of malaria, there is paucity of information on KAP among women in the reproductive age of 15–49 years in Burkina Faso. Therefore, the objective of this study is to understand women’s knowledge, attitudes and practices of malaria and its control in order to create a synergy between community efforts and governmental/nongovernmental malaria control interventions in Burkina Faso.

## Methods

### Study area and survey

This study used secondary data from the 2014 Burkina Faso Malaria Indicator Survey (MIS). We accessed the data from MEASURE DHS database at http://dhsprogram.com/what-we-do/survey/survey-display-481.cfm. The survey was conducted by the Burkina Faso National Statistical Agency as part of the International Demographic and Health Survey program known as MEASURE DHS, which is currently active in 90 countries and conducted under the auspices of the United States Agency for International Development (USAID) with the technical assistance of ICF International, based in the USA. The Demographic and Health Surveys (DHSs) are free, public datasets, though researchers must register with MEASURE DHS and submit a request before access to DHS data is granted. This data request system ensures that all users understand and agree to basic data usage ethics standards.

The Burkina Faso Malaria Indicator Survey (MIS) 2014 is a cross-sectional, nationally representative survey following RBM Monitoring and Evaluation Reference Group (MERG) guidelines. The MIS was conducted from 29 September to 28 November 2014 and provides information on the knowledge and practice of malaria prevention in Burkina Faso, and is part of phase 7 of the Demographic and Health Survey series (DHS). In brief, 8111 women ages 15–49 from 6,448 households were successfully interviewed. The details of the survey and sampling procedures have been described in the final report [30].

### Selection of variables

In this study, we assessed women’s knowledge about 1) preventive measures, 2) causes and 3) symptoms of malaria, as well as malaria prevention practices for their children and during pregnancy. The MIS 2014 included a number of basic questions for this purpose which are described below:

- Knowledge about preventive measures: 1) Sleeping under a mosquito net, 2) Sleeping under a mosquito net impregnated with insecticide, 3) Using insecticide sprays, creams, lotions, 4) Taking preventative medications, 5) Using insecticide coils, 6) Decoction/plant juice/Root to drink as a preventive measure, 7) Making sure surroundings are clean, 8) Using a coil smoke, 9) Covering the body.
- Knowledge about causes: 1) Mosquito bite, 2) Heavy oil consumption, 3) Work-related fatigue, 4) Insufficient sleeping, 5) Direct exposure to sunlight, 6) Consumption of mangoes / sweet fruits 7) Milk consumption 8) Drinking dirty water.
- Knowledge about common symptoms of malaria: 1) Fever 2) Vomiting and lack of appetite 3) High temperature with convulsions 4) High temperature with fainting 5) Persistent fever 6) Convulsions 7) Jaundice 8) Headache 9) Urine.

The socio-demographic characteristics considered were: Age (15–24, 25–34, 35–44, 44+), Religion (Islam, Christian, Other), Education (No education, Primary, Secondary, Higher), Wealth index (Poorest, Poorer, Middle, Richer, Richest), Number of household (HH) members (6, >6), Sex of household head (Male, Female), Household possession of radio (No, Yes), TV (No, Yes), Received antenatal care.

### Data analysis

Data analysis was conducted with STATA version 14. Basic social and demographic characteristics were presented as percentages and frequencies. Analyses were stratified by area of residence (urban/rural) due to the influence of neighbourhood status on health knowledge, self-efficacy and practice. Chi-square tests were employed to assess any significant difference in knowledge of prevention, causes, symptoms, and practices between urban and rural areas. Logistic regression was used to examine the factors associated with knowledge of malaria among women in Burkina Faso. The level of significance was set at p<0.05.

## Ethics statement

Before each interview, all participants gave informed consent to take part in the survey. The DHS Program maintains strict standards for ensuring data anonymity and protecting the privacy of all participants. ICF International ensures that the survey complies with the U.S. Department of Health and Human Services regulations for the protection of human subjects, whilst the host country ensures that the survey complies with local laws and norms. Further approval for this study was not required since the data is secondary and is available in the public domain. More details regarding DHS data and ethical standards are available at: http://goo.gl/ny8T6X.

## Results

### Descriptive analysis

A total of 8,111 women aged 15 to 49 years were included in the study. Basic socio-demographic characteristics of the participants were presented as frequencies and percentages in Table 1. Mean age was 28.63 years (SD 9.41). Table 1 shows that most of the women were aged 15 to 19 years (21.1%), prominent faith was Islam (64.3%) and about two-third had no formal education (71.3%). About half of the respondents had low economic status. A majority of the households had more than six members (57.7%). Male-headed households were predominant (90.4%) in the sample. Approximately three-fifths of the women had a radio (61.1%) and only about one-fifth had television (20.9%). The uptake of antenatal care (ANC) services was low, only about 14% of the women reported receiving any ANC. There was a significant association between place of residence and socio-economic and maternal factors in certain variables among women.

**Table 1:** Characteristics of the women (Burkina Faso Malaria Indicator Survey 2014).

Variable	Total		Urban	Rural	P
**Age (28.63±9.41)**	n = 8,111	%	20.6	79.4	0.128
15–19	1714	21.1	22.1	20.9	
20–24	1411	17.4	15.8	17.8	
25–29	1417	17.5	16.4	17.7	
30–34	1197	14.8	14.1	14.9	
35–39	1065	13.1	14.0	12.9	
40–44	738	9.1	9.9	8.9	
45–49	569	7.0	7.7	6.8	
**Religion**					<0.001*
Islam	5212	64.3	65.1	64.0	
Christianity	2222	27.4	33.0	25.9	
Other	677	8.3	1.9	10.0	
**Education**					0.069
No education	5781	71.3	69.0	71.9	
Primary	1109	13.7	14.7	13.4	
Secondary	1154	14.2	15.2	14.0	
Higher	67	0.8	1.1	0.7	
**Wealth index**					0.017*
Poorest	1630	20.1	23.1	19.3	
Poorer	1678	20.7	19.8	20.9	
Middle	1767	21.8	21.1	22.0	
Richer	1734	21.4	21.0	21.5	
Richest	1302	16.1	15.0	16.3	
**No. of HH members**					0.331
6	3428	42.3	41.8	42.4	
>6	4683	57.7	58.2	57.6	
**Sex of HH head**					<0.001*
Male	7335	90.4	92.6	89.9	
Female	776	9.6	7.4	10.1	
**HH has radio**					<0.001*
No	3066	37.9	28.8	40.1	
Yes	4944	61.1	71.2	59.9	
**HH has TV**					<0.001*
No	6314	78.1	38.9	87.9	
Yes	1687	20.9	61.1	12.1	
**Received ANC**					0.005*
No	6977	86.0	88.0	85.5	
Yes	1134	14.0	12.0	14.5	

*significant at p<0.05; N.B. ANC was counted as ‘Yes’ when offered by health professionals e.g. doctor, nurse or community health worker.

The assessment of accurate knowledge of malaria prevention, causes and symptoms are shown in Table 2. A majority of the women (97.4%) and over 80% of the women reported that sleeping under a mosquito net and sleeping under insecticide treated net respectively, are the best practices to prevent malaria. Furthermore, a very low proportion of the women opined that: using insecticide sprays, creams and lotions (6.1%), taking preventative medications (6.4%), insecticide coils (4.5%), drinking plant juice/root (5.9%), coil smoke (4.9%) and covering the body (8.7%) were the best preventive measures. About one-fifth reported that keeping the surrounding clean is the best preventive measure.

**Table 2:** Knowledge about best preventive measures, main causes and symptoms of malaria (n = 8111).

Item	Rural (%)	Urban (%)	Total (%)
**Best way to prevent malaria**			
Sleep under a mosquito net	97.2	98.0	97.4
Sleep under a mosquito net impregnated with insecticide	80.6	80.8	80.7
Using insecticide spray, creams, lotions	94.4	92.3	93.9
Take preventive medications	93.3	94.5	93.6
Using insecticide coil	95.4	96.0	95.5
Decoction/plant juice/root to drink as a preventive	93.9	94.8	94.1
Making sure surroundings are clean	80.4	77.5	79.8
Using a coil smoke	95.1	95.0	95.1
Cover the body	91.1	92.3	91.3
**Main cause of malaria**			
Mosquito bite	81.4	100.0	85.2
Heavy oil consumption	98.1	96.2	97.7
Work-related fatigue	96.5	97.4	96.7
Insufficient sleeping	98.9	99.9	99.1
Direct exposure to sunlight	92.8	97.3	93.8
Consumption of mangoes/sweet fruits	97.3	99.7	97.8
Milk consumption	99.0	99.9	99.1
Dirty water	92.8	54.0	84.9
**Symptoms of malaria**			
Fever	76.7	100.0	81.5
Vomiting, lack of appetite	46.8	84.3	54.5
High temperature with convulsion	94.1	94.5	94.2
High temperature with fainting	97.1	99.0	97.5
Persistent fever	96.3	99.7	97.0
Convulsion	92.2	98.4	93.5
Jaundice	87.4	97.7	89.5
Headache	74.8	59.2	71.6
Urine	98.5	98.1	98.4

In addition, a majority of the women (85.2%) knew that mosquito bites could cause malaria. While low proportion accepted that heavy oil consumption (2.3%), work-related fatigue (3.3%), insufficient sleeping (0.9%), exposure to sunlight (6.2%), consumption of certain fruits (2.2%), milk (0.9%), and dirty water (15.1%) can be the main cause of malaria.

More so, a majority of the women identified fever (81.5%), vomiting (54.5%) as the main symptoms of malaria. About 10% reported jaundice and 28.4% headache as the common symptoms. Those who reported other physical symptoms, such as fainting, convulsion, and urinary problems were comparatively lower.

On the malaria prevention practices, out of 4,656 women, 20.4% reported not using any net at all and 77.9% reported using only insecticide treated nets for children during the night before the survey as shown in Table 3. Regarding the use antimalarial drugs for febrile illnesses during malaria, amodiaquine was the commonest one (21.6%) followed by fansidar, quinine and chloroquine. A majority of the women (84.7%) reported taking antimalarial drugs during pregnancy. No significant different was observed in these practices between urban and rural areas except for taking fansidar.

**Table 3:** Prevalence of malaria prevention practices (Burkina Faso Malaria Indicator Survey 2014).

Types of practices	Total	Urban (%)	Rural (%)	p
Type of mosquito bed net(s) child slept under last night(n = 4645)				0.599
No net	20.4	20.9	20.4	
Only treated nets	77.9	77.8	77.9	
Only untreated nets	1.7	1.3	1.7	
Fansidar taken for fever/cough (n = 2032)				0.012*
No	88.6	85.2	89.5	
Yes	11.4	14.8	10.5	
Chloroquine taken for fever/cough(n = 2032)				0.379
No	99.0	98.7	99.0	
Yes	1.0	1.3	1.0	
Amodiaquine taken for fever/cough(n = 2032)				0.397
No	78.4	79.0	78.2	
Yes	21.6	21.0	21.8	
Quinine taken for fever/cough(n = 2032)				0.309
No	98.8	98.5	98.9	
Yes	1.2	1.5	1.1	
During this pregnancy did you take medicine to prevent malaria? (n = 5130)				0.427
No	15.3	14.8	15.4	
Yes	84.7	85.2	84.6	

*significant at p<0.001

**Fig 1:**
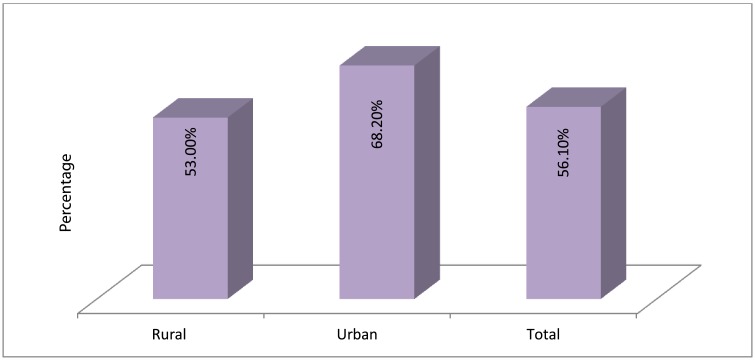
Level of accurate knowledge of malaria.

Fig 1 showed that the level of the knowledge of malaria preventive measures, causes and symptoms was low. The level of knowledge of malaria for rural dwellers was at 53%, while the urban dwellers had a higher level of knowledge at 68.2%. In sum, there was 56.1% level of the accurate knowledge of malaria preventive measures, causes and symptoms among women of reproductive age in Burkina-Faso.

The association between the level of knowledge on malaria and socio-demographic and maternal factors was tested using Chi-squared bivariate analysis to determine the variables that will be included in the logistic regression model. Results from Table 4 revealed that place of residence, religion, educational attainment; ANC and being in possession of radio or television had signification association with the level of knowledge. On the contrary, age, wealth index, sex of household head and source of antimalarial drug during pregnancy, did not show any significant association with the level of knowledge among women aged 15–49 years.

**Table 4:** Socio-demographic and maternal characteristics associated with knowledge of malaria (n = 8111).

Variable	Knowledge of malaria		Total	P-value
	Accurate	Poor		
**Age (years)**				
15–19	1005(58.6)	709(41.4)	1714(100.0)	0.175
20–24	794(56.3)	617(43.7)	1411(100.0)	
25–29	760(53.6)	657(46.4)	1417(100.0)	
30–34	672(56.1)	525(43.9)	1197(100.0)	
35–39	596(56.0)	469(44.0)	1065(100.0)	
40–44	418(56.6)	320(43.4)	738(100.0)	
45–49	308(54.1)	261(45.9)	569(100.0)	
**Place of residence**				
Urban	1139(68.2)	530(31.8)	1669(100.0)	<0.001*
Rural	3414(53.0)	3028(47.0)	6442(100.0)	
**Religion**				
Islam	3014(57.8)	2198(42.2)	5212(100.0)	0.001*
Christianity	1539(53.1)	1360(46.9)	2899(100.0)	
**Highest level of education**				
No formal education	3161(54.7)	2620(45.3)	5781(100.0)	<0.001*
Primary	635(57.3)	474(42.7)	1109(100.0)	
Secondary	711(61.6)	443(38.4)	1154(100.0)	
Higher	46(68.7)	21(31.3)	67(100.0)	
**Wealth index**				
Poorest	905(55.5)	725(44.5)	1630(100.0)	0.914
Poorer	939(56.0)	739(44.0)	1678(100.0)	
Middle	1009(57.1)	758(42.9)	1767(100.0)	
Richer	972(56.1)	762(43.9)	1734(100.0)	
Richest	728(55.9)	574(44.1)	1302(100.0)	
**Sex of household head**				
Male	4099(55.9)	3236(44.1)	7335(100.0)	0.162
Female	454(58.5)	322(41.5)	776(100.0)	
**Radio**				
Yes	3275(64.9)	1770(35.1)	5045(100.0)	<0.001*
No	1278(41.7)	1788(58.3)	3066(100.0)	
**Television**				
Yes	1214(67.6)	583(32.4)	1797(100.0)	<0.001*
No	3339(52.9)	2975(47.1)	6314(100.0)	
**Received ANC**				
Yes	896(79.0)	238(21.0)	1134(100.0)	<0.001*
No	3657(52.4)	3320(47.6)	6977(100.0)	
**Source of antimalarial during pregnancy**				
Antenatal visit	1910(55.5)	1533(44.5)	3443(100.0)	0.895
Another facility visit	10(52.6)	9(47.4)	19(100.0)	
Other source	5(62.5)	3(37.5)	8(100.0)	

*significant at p<0.05

Results from Table 5 revealed that women in rural locations had 40% reduction in the odds of having accurate knowledge of malaria when compared to the urban women (aOR = 0.60; 95%CI: 0.52–0.68). For religion, it was reported that Christianity had 14% reduction in the odds of having accurate knowledge when compared to Islam (aOR = 0.86; 95%CI: 0.78–0.95). Educational attainment was a factor of the knowledge of malaria. The odds of having accurate knowledge of malaria increased as the educational level increased; hence, secondary and higher education had 29% and 93% increase in the odds of having accurate knowledge of malaria when compared to the women without formal education. Furthermore, respondents who had radio and television were 2.59 times and 1.22 times more likely to have accurate knowledge of malaria when compared to those who have none. It was also evident that antenatal care services teach women about malaria as women who reportedly utilized ANC services were 3.90 times more likely to have accurate knowledge of malaria when compared to those who did not utilize skilled ANC services (aOR = 3.90; 95%CI = 3.34–4.56).

**Table 5:** Multivariable analysis of the factors associated with knowledge of malaria.

Variable	Adjusted Odds Ratio(95%CI)	P-value
**Place of residence**		
Urban(ref)	1.00	
Rural	0.60(0.52–0.68)	<0.001*
**Religion**		
Islam(ref)	1.00	
Christianity	0.86(0.78–0.95)	0.003*
**Highest level of education**		
No formal education(ref)	1.00	
Primary	1.07(0.93–1.23)	0.344
Secondary	1.29(1.13–1.48)	<0.001*
Higher	1.93(1.13–3.32)	0.017*
**Radio**		
No(ref)	1.00	
Yes	2.59(2.35–2.86)	<0.001*
**Television**		
No(ref)	1.00	
Yes	1.22(1.07–1.39)	0.003*
**Received ANC**		
No (ref)	1.00	
Yes	3.90(3.34–4.56)	<0.001*

*significant at p<0.05; Pseudo R-squared = 0.08

## Discussion

Knowledge about Malaria was analysed across four domains: prevention, causes, symptoms and preventive practises pursued. In the domain of prevention, majority of the women (97.4) reported that sleeping under a mosquito net and most of the women (80%) reported that sleeping under an insecticide treated net is the best practice to prevent malaria. A small proportion of women affirmed that using other preventive measures like insecticide sprays, using creams, lotions or keeping the surrounding clean can help to prevent transmission of malaria. Overall it can be stated that the knowledge about prevention, causes and symptoms of malaria was slightly above average among the respondents. This finding is similar to findings from previous studies where knowledge about preventive measures was found to be relatively high [31, 32]. One study interestingly reported that although the knowledge of preventive measures was high, it does not translate into preventive practices [31]. This was an important finding during our analysis and will be further discussed in detail along with the preventive practices pursued among women. The finding about better knowledge of preventive practices might be illustrative of a positive impact of behavioural communication techniques used toward malaria control.

In general, there was a difference in the knowledge of malaria between rural and urban locations. For the knowledge items, urban women were more aware that malaria could be caused by mosquito bites. Moreover, rural dwellers indicated more of the unrelated factors for malaria, such as heavy oil consumption, work related fatigue, insufficient sleeping, and exposure to sunlight, consumption of certain fruits, milk and dirty water as causes of malaria. The findings may be presumed to be related to a myriad of other factors, such as level of education in rural areas and access to media or behavioural communication at ANC. For example, one study reported significant association between level of education and the knowledge about transmission of malaria in rural areas [33]. Overall, previous studies have reported findings similar to the current study in that, urban areas have significantly higher knowledge about the causes of malaria [33, 34].

Fever and vomiting were identified as the most common symptoms of malaria. Nearly 10% and 28% reported symptoms such as jaundice and headache respectively. However, it is noteworthy that respondents from urban areas were more knowledgeable than those from rural areas and the difference was statistically significant. This finding is again consistent with findings from previous studies [31, 32, 33, 34]. The findings suggest that except for the knowledge about prevention of malaria, the urban areas fared better in terms of knowledge about causes and symptoms of malaria than the rural areas. Notably, rural participants had better knowledge of several symptoms (with few exceptions), especially that of jaundice. Consistent with the findings from a previous study, the current study also inclines to the conclusion that there is dearth of information percolating to the rural areas in terms of control strategies for malaria. This dearth may be directly related to the communication strategies employed and/or could be consequential to a number of related factors like education level, local cultural practices, media exposure and others. Interestingly, the message about preventive methods is reaching the rural population, however, that about causes and symptoms is not. This is an area that needs further research.

Another aspect of the current study is studying the preventive practices pursued. Knowing about the prevention methods, causes and symptoms of malaria is one thing and employing preventive practices is another. For example, 97.4% (n = 8111) of women in the current study indicated knowing that sleeping under a net is the best practice to prevent malaria. Despite this, about one-fifth of the women (20.4%, n = 4645) reported not using any bed net for children during the night before the survey. This is true for both urban and rural areas. So the messages for malaria control which appeared well percolated in both urban and rural areas are still not pragmatically useful. Similarly, one study reported that generic community education programs may not be as effective in endemic areas [35]. This indicates the need for more integrated approaches which target not only communicating the current best practices in malaria prevention and control but also customising these efforts to the local cultural practices which prevent the pragmatic uptake of such messages. This remains true for both urban and rural areas.

### Strength and limitations

This study comprised of a nationally representative large dataset. More so, there was high response among the study participants. Nonetheless, the current study has certain limitations in terms of the availability of data. The current study relying on the data available in public domain had no control over the quality and type of data. One potentially conflicting observation was the high prevalence of antimalarial drug use during pregnancy despite the fact that the rate of ANC attendance was notably low. This is perhaps due to the presence of traditional service providers who are usually not considered when measuring the prevalence of skilled care providers for reproductive health. Finally, the prevalence of utilization of antimalarial drugs could be higher had a broader range of medications available in the market be considered [36].

## Conclusion

The findings indicate that the general knowledge of malaria could be improved upon by both urban and rural dwellers. There were statistically significant differences observed between urban and rural areas in terms of the knowledge about the prevention, causes and symptoms of malaria with the urban areas faring better than the rural areas. Also, there were factors, such as level of education, place of residence, access to the media, ANC and religion, that were associated with the level of knowledge of malaria. Overall, it can be stated that there is limited knowledge of the best practices in malaria prevention and control. The knowledge of malaria must be improved and translated into good practices to enhance prevention and control. Several factors, such as education level, exposure to the media, ANC, religion and place of residence, need to be integrated into the current generalized communication strategies employed by the ministry of health to improve the prevention and control of malaria. There is need to conduct more evidence-based research on the role of socio-cultural practices among women toward malaria prevention and control.

## Abbreviations

ACT: Artemisinin-based Combined Therapy
IRS: Indoor Residual Spraying
ITN: Insecticide Treated bed Nets
KAP: Knowledge, Attitudes and Practices
SMC: Seasonal Malaria Chemoprevention
